# Patterns of meniscal tears in multiligament knee injuries with and without dislocation: a retrospective study from a Level I trauma center

**DOI:** 10.1186/s43019-026-00307-7

**Published:** 2026-01-27

**Authors:** Luis Henrique Longo, Bruno Dada Gulini, Marcos Paulo Tercziany Vanzin, Fernando Martins Rosa, Luca Eiji Sohn Sato, Luis Antonio de Ridder Bauer

**Affiliations:** Knee Surgery Group, Department of Orthopaedic Surgery, Complexo Hospitalar do Trabalhador, Curitiba, Brazil

**Keywords:** Multiligament knee injury, Knee dislocation, Meniscal tear, Magnetic resonance imaging

## Abstract

**Background:**

Multiligament knee injuries (MLKI) are rare but severe, often associated with knee dislocation and carry a high risk of neurovascular complications. Although ligamentous reconstruction has been widely studied, there is limited evidence addressing the incidence and specific patterns of meniscal injuries in this setting.

**Methods:**

We retrospectively analyzed skeletally mature patients admitted to a Level I trauma center between January 2022 and December 2024 with MLKI, with or without knee dislocation. Demographics, trauma mechanism, ligamentous, and meniscal injury patterns were reviewed on the basis of magnetic resonance imaging (MRI) and surgical records. Meniscal tears were classified by location and morphology. Statistical analysis included Fisher’s exact test, chi-square tests, and univariate logistic regression with significance set at *p* < 0.05.

**Results:**

A total of patients were included: 35 (63.6%) with knee dislocation and 20 (36.4%) without. Mean age was 36.8 ± 13.2 years, and 81.8% were male. Magnetic resonance imaging was performed at a mean of 28.1 ± 7.3 days after injury. High-energy trauma accounted for 69.1% of cases and was significantly associated with dislocation (*p* = 0.001). Medial meniscus tears in patients with dislocation were predominantly radial/complex/oblique (72.7% versus 0% in nondislocated, *p* = 0.004; OR not calculable due to perfect separation), while all nondislocated patients had longitudinal tears. Lateral meniscus tears showed a similar pattern, with radial/complex/oblique tears more frequent in dislocation (80.0% versus 20.0%, OR = 16.00, 95% CI 1.27–200.92, *p* = 0.031).

**Conclusions:**

Knee dislocations demonstrate distinct meniscal tear patterns compared with nondislocated MLKI, with radial and complex tears predominating in dislocated knees. Recognition of these differences may assist in accurate diagnosis and individualized surgical planning.

## Introduction

Multiligament knee injuries (MLKI) are defined as the rupture of at least two of the four primary stabilizing ligaments: the anterior cruciate ligament, posterior cruciate ligament, medial collateral ligament, and posterolateral corner [1, 2]. The term is often used interchangeably with knee dislocation; however, the latter is characterized by a multiligament injury associated with loss of tibiofemoral joint congruence [1, 3].

Knee dislocations represent severe injuries that may threaten limb integrity, potentially resulting in permanent sequelae or even amputation [4, 5]. Although rare, with an estimated incidence of 0.2% and accounting for approximately 0.5% of all joint dislocations, these numbers are likely underestimated, since many cases may spontaneously reduce and, consequently, remain undocumented at hospital admission [6]. Accurate recognition of these injuries is crucial, as knee dislocations carry an increased risk of acute compartment syndrome and are associated with vascular complications in up to 18% and neurological complications in approximately 25% of patients [1, 7].

Knee dislocations are commonly classified using the Schenck system [8], which categorizes injuries on the basis of involved ligamentous structures and has been extended to describe multiligament injuries without dislocation [3, 9].

Meniscal injuries are frequently associated with MLKI and may significantly influence functional outcomes and surgical planning [10–12]. The morphological pattern of meniscal tears—whether longitudinal, radial, complex, or root—directly affects repairability, surgical technique selection, and long-term prognosis [13]. Radial and complex tears, for instance, are less amenable to repair and may require specific fixation techniques or partial meniscectomy, whereas longitudinal tears are often repairable with better functional preservation [14]. Furthermore, unrecognized or inadequately treated meniscal injuries in the setting of MLKI reconstruction may lead to persistent instability, accelerated cartilage degeneration, and suboptimal clinical outcomes [15].

Despite the clinical relevance of meniscal injuries in MLKI, there is limited evidence specifically comparing tear patterns between patients with and without knee dislocation. Most studies have focused on ligamentous reconstruction without detailed characterization of meniscal morphology or stratification by dislocation status. Whether the biomechanical forces associated with frank dislocation result in distinct meniscal tear patterns compared with nondislocated MLKI remains unclear. Understanding these patterns may enhance diagnostic accuracy, guide arthroscopic survey protocols, and inform individualized surgical planning [16].

In this context, the purpose of the present study was to compare the incidence and patterns of associated meniscal injuries in patients with multiligament knee injuries, distinguishing those with and without joint dislocation. We hypothesized that: (1) knee dislocations would be associated with a higher incidence of meniscal tears compared with nondislocated MLKI; (2) the morphological pattern of meniscal tears would differ between dislocated and nondislocated knees, with radial and complex tears predominating in dislocations and longitudinal tears in nondislocated injuries; and (3) high-energy trauma mechanisms would be more strongly associated with knee dislocation and complex meniscal injury patterns.

## Materials and methods

This retrospective study included patients admitted to a Level I trauma center between January 2022 and December 2024 with a diagnosis of multiligament knee injury (MLKI), with or without knee dislocation. All cases were initially assessed by the hospital trauma team following the Advanced Trauma Life Support (ATLS) protocol [17].

**Fig. 1:**
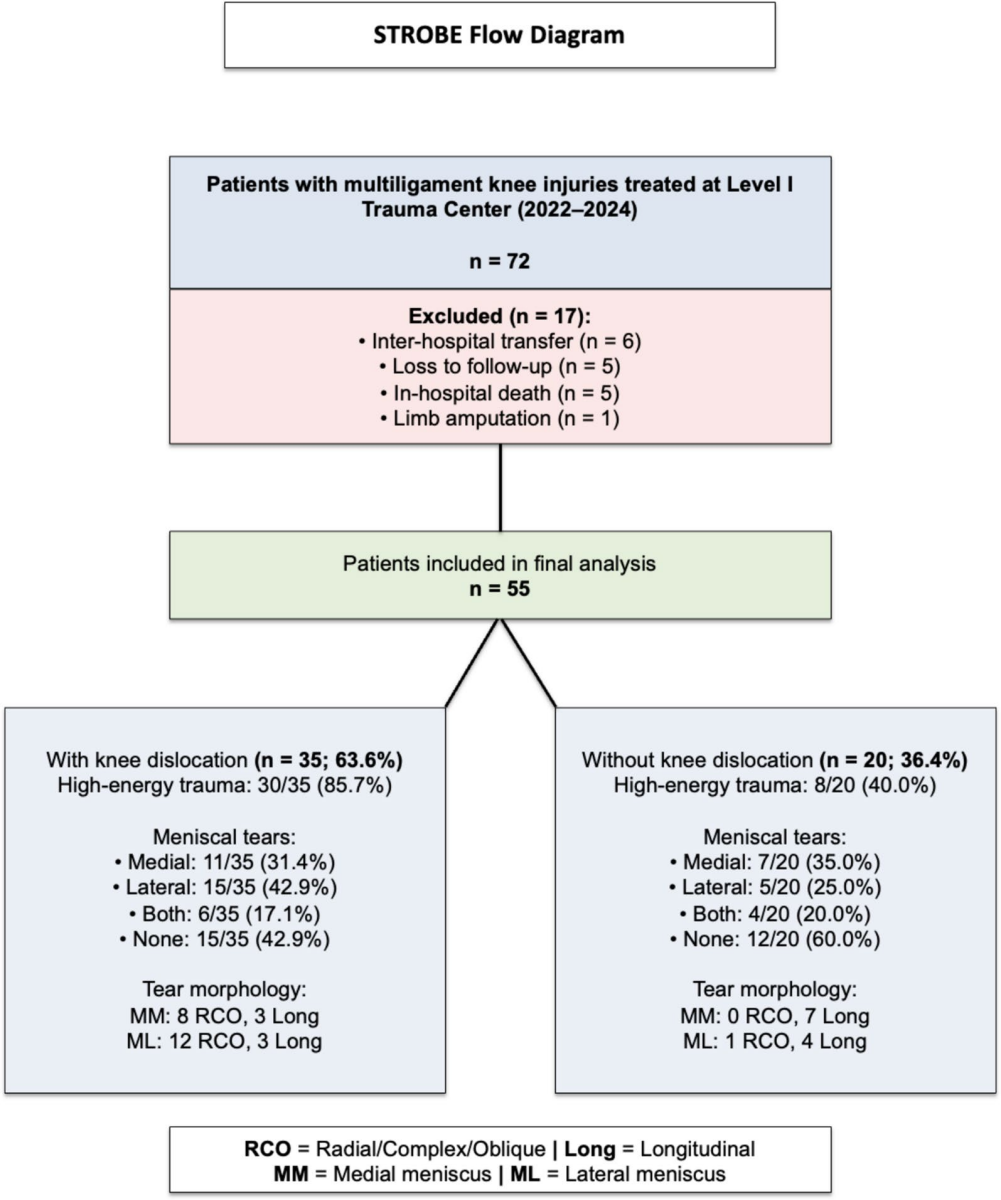
STROBE flow diagram

A total of 72 patients were initially identified. Inclusion criteria comprised skeletally mature individuals with either documented loss of tibiofemoral joint congruence, consistent with knee dislocation, or rupture of at least two of the four primary stabilizing ligaments—the anterior cruciate ligament (ACL), posterior cruciate ligament (PCL), medial collateral ligament (MCL), and posterolateral corner (PLC)—confirmed on magnetic resonance imaging (MRI) and described by a musculoskeletal radiologist. Exclusion criteria included prior knee injury or surgery, incomplete data, loss to follow-up (*n* = 5), inter-hospital transfer (*n* = 6), limb amputation (*n* = 1), or in-hospital death (*n* = 5). After applying these criteria, 55 patients were included in the final analysis. A flow diagram summarizing patient inclusion and exclusion is presented in Fig. 1.

Knee dislocation was defined as a documented loss of tibiofemoral congruence on radiographs or clinical examination, or evidence of spontaneous reduction with a characteristic injury pattern on MRI (complete rupture of at least three major ligaments, including both cruciates).

High-energy trauma included motor vehicle and motorcycle collisions, pedestrian accidents, and falls from heights greater than 2 m, whereas low-energy trauma encompassed ground-level falls, sports injuries, and torsional mechanisms.

As part of the institutional management protocol, patients with knee dislocation initially underwent transarticular external fixation for 3–4 weeks to allow soft tissue recovery and reduction of acute inflammation. Following removal of the fixator, MRI was performed.

Patients without dislocation were discharged and underwent MRI either before or shortly after the first outpatient visit, typically within 1–6 weeks of injury (most commonly at 2 weeks). Overall, MRI was performed at a mean of 28.1 ± 7.3 days after injury (median 29.0 days, range 14–45 days), with 90.9% of examinations conducted in the subacute phase (> 14 days).

All patients underwent MRI following clinical stabilization. Examinations were performed using a Signa Explorer 1.5 T scanner (GE Healthcare, Milwaukee, WI, USA), with the knee positioned in approximately 15° of flexion. Images were acquired in sagittal, coronal, and axial planes, with 4.5-mm slice thickness and 0.5-mm interslice gap. The field of view was set to 160 mm in all planes. Images were exported in DICOM format via the Arya system (Pixeon, São Caetano do Sul, Brazil) and subsequently reviewed by both a radiologist and an orthopedic surgeon.

The MRI protocol included T2-weighted fast spin-echo (FSE) sequences in sagittal and coronal planes, which provide optimal contrast for evaluating meniscal morphology, signal changes, and associated soft-tissue injuries. An axial T2-weighted or proton-density fat-suppressed (PD-FS) sequence was used to assess cruciate ligaments and periarticular structures. In addition, T1-weighted sagittal sequences were obtained to evaluate osseous structures and intra-articular loose bodies, given their superior ability to differentiate bone marrow and fat-containing tissues. It is acknowledged that the 4.5-mm slice thickness may have limited sensitivity for detecting small radial tears or subtle root pathology compared with thinner-slice protocols.

MRI scans were independently analyzed by a musculoskeletal radiologist and a fellowship-trained knee surgeon. No formal inter-reader reliability coefficient (kappa) was calculated; however, any discrepancies were resolved by consensus through joint image review, ensuring consistent classification of ligamentous and meniscal injuries.

Meniscal lesions were evaluated according to topography (anterior horn, body, or posterior horn) and classified by morphological pattern (longitudinal, horizontal, oblique, radial, complex, or bucket-handle) on the basis of O’Connor’s criteria [18]. Ligamentous structures were categorized as intact, partially torn, or completely ruptured, and grouped into five categories: ACL, PCL, MCL, LCL, and PLC (including the LCL, popliteus tendon, and popliteofibular ligament).

Associated fractures and neurovascular injuries were also documented.

Most patients subsequently underwent surgical treatment for ligamentous reconstruction; however, the exact proportion of operated cases and arthroscopic confirmation of meniscal findings were not systematically recorded in the database and therefore were not included in this analysis. The study was designed as a radiology-based investigation focused on MRI-detected meniscal morphology.

Collected data were organized in Google Sheets^**®**^ and analyzed using IBM SPSS Statistics, version 28.0 (IBM Corp., Armonk, NY, USA). Categorical variables were compared using chi-square tests or Fisher’s exact test when expected cell frequencies were < 5. Continuous variables were compared using Student’s *t*-test or the Mann–Whitney *U* test**,** as appropriate.

When feasible, univariate logistic regression was performed with meniscal tear morphology (radial/complex/oblique versus longitudinal) as the outcome and dislocation status as the primary predictor. Perfect separation in the medial meniscus dataset precluded calculation of odds ratios (OR) via standard regression; thus, Fisher’s exact test was used as the main inferential method. For the lateral meniscus, where separation did not occur, ORs with 95% confidence intervals (CIs) were reported. A post-hoc power analysis was conducted using Cohen’s *h* to estimate effect size and confirm the adequacy of the sample size for detecting the observed effects. Statistical significance was defined as *p* < 0.05**.**

This study was approved by the Institutional Research Ethics Committee (CAAE: 88220725.5.0000.5225).

## Results

A total of 55 patients with multiligament knee injuries admitted between January 2022 and December 2024 were included in the analysis. Of these, 35 (63.6%) presented with associated knee dislocation, whereas 20 (36.4%) had multiligament injury without dislocation. The right knee was affected in 58.2% of cases and the left knee in 41.8%, with no bilateral involvement observed.

Most patients were male (*n* = 45; 81.8%), while 10 (18.2%) were female. The mean age was 36.8 ± 13.2 years (range, 18–72). No statistically significant differences were found between the dislocation and nondislocation groups regarding age (*p* = 0.542) or sex distribution (*p* = 0.461).

Motorcycle collisions were the leading mechanism of injury, accounting for more than half of all cases (50.9%), followed by torsional trauma (23.6%) and ground-level falls (10.9%). Other mechanisms included pedestrian accidents (*n* = 3), bicycle falls (*n* = 2), falls from height (*n* = 1), assaults (*n* = 1), and skateboard accidents (*n* = 1), each representing fewer than 6% of cases. When grouped by trauma energy, high-energy mechanisms accounted for 69.1% of injuries (*n* = 38) and were significantly associated with knee dislocation (*p* = 0.001). Table 1 summarizes the baseline characteristics of the study population.

**Table 1:** Baseline characteristics of patients with multiligament knee injuries (*n* = 55)

Variable	With dislocation (*n* = 35)	Without dislocation (*n* = 20)	Total (*n* = 55)	*p*-value	95% CI (difference)
Age, mean ± SD (years)	37.2 ± 12.9	36.1 ± 13.8	36.8 ± 13.2	0.542 †	−6.3 to 8.5
Male sex, *n* (%)	29 (82.9)	16 (80.0)	45 (81.8)	0.461 ‡	–
High-energy trauma, *n* (%)	30 (85.7)	8 (40.0)	38 (69.1)	0.001 ‡	24.3–66.2

^†^Independent-samples *t* test

^‡^ Chi-square or Fisher’s exact test, as appropriate

Several associated injuries were identified in this cohort. The most frequent was peroneal nerve injury, observed in five patients (9.1%), followed by tibial plateau fractures in four patients (7.3%). Distal femoral fractures and popliteal artery injuries each occurred in two patients (3.6%). Less common findings included patellar fracture and patellar tendon rupture, each present in one patient (1.8%).

Based on MRI findings, complete ruptures of the main stabilizing ligaments were observed as follows: the anterior cruciate ligament (ACL) in 76.4% of cases (42 of 55 knees), the posterior cruciate ligament (PCL) in 67.3% (37 knees), the lateral collateral ligament (LCL) in 47.3% (26 knees), the medial collateral ligament (MCL) in 34.5% (19 knees), and the posterolateral corner (PLC) structures in 25.5% (14 knees).

The distribution of ligament injury patterns according to the Schenck classification, as well as their association with the presence of meniscal tears, is summarized in Table 2.

**Table 2:** Schenck classification of ligament injury patterns and meniscal involvement

Classification/Injury Schenck classification	*n* (%)	Meniscal tear *n* (%)
KD I–ACL or PCL + collateral	14 (25.5%)	6 (42.9%)
KD II–ACL + PCL	2 (3.6%)	1 (50.0%)
KD III-L–ACL + PCL + LCL/PLC	9 (16.4%)	2 (22.2%)
KD III-M–ACL + PCL + MCL	8 (14.5%)	3 (37.5%)
KD IV–ACL + PCL + MCL + LCL/PLC	15 (27.3%)	9 (60.0%)
KD V–Periarticular fracture	7 (12.7%)	3 (42.9%)

Among the 55 patients included in the study, 18 medial and 20 lateral meniscal tears were identified. In the knee dislocation group, the incidence of meniscal injury was 31.4% (11 of 35 knees) for the medial meniscus and 42.8% (15 of 35 knees) for the lateral meniscus. In patients without dislocation, the incidence was 35.0% (7 of 20 knees) for medial and 25.0% (5 of 20 knees) for lateral meniscal tears.

**Fig. 2:**
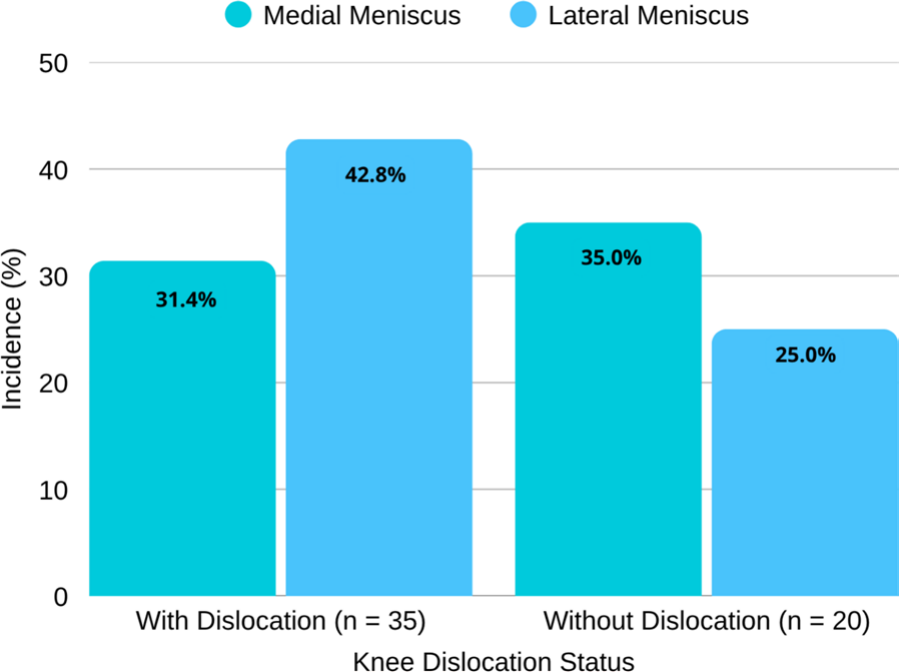
Meniscal tear incidence by dislocation status

These distributions are illustrated in Fig. 2, which demonstrates the relative frequency of medial and lateral meniscal involvement according to dislocation status.

**Fig. 3:**
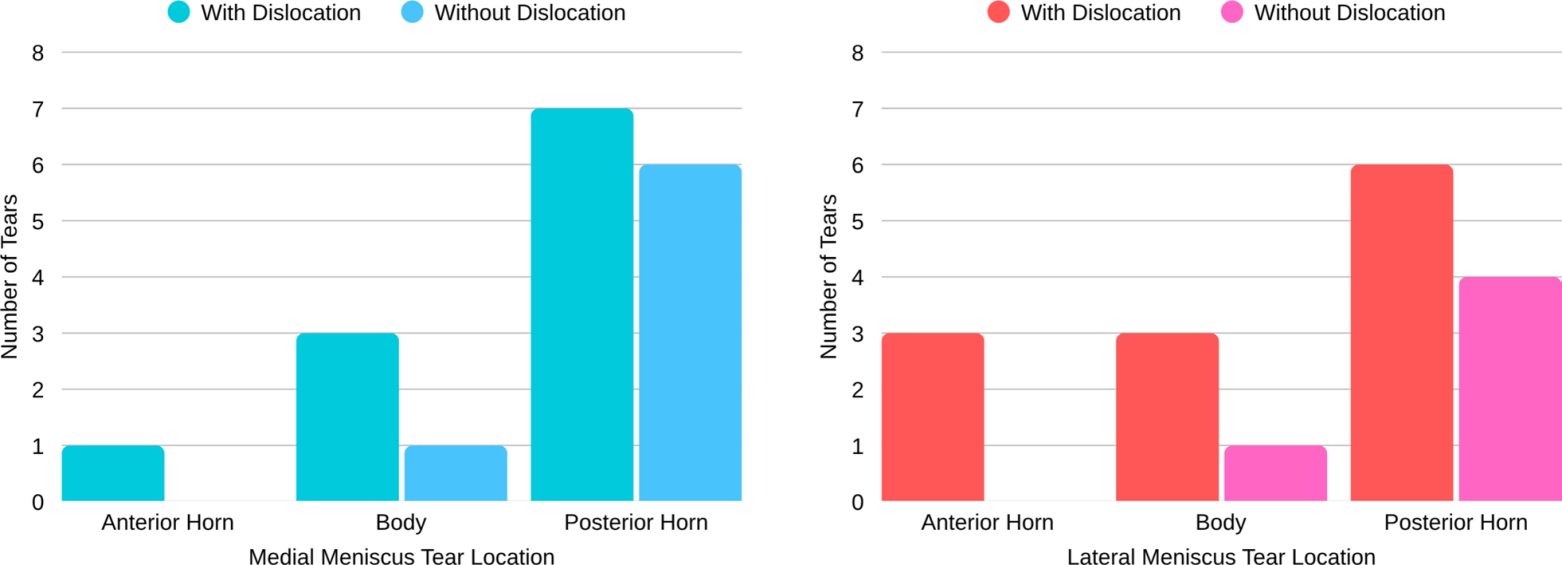
Topographic distribution of medial (left) and lateral (right) meniscal tears according to dislocation status

For the medial meniscus, patients with dislocation showed a predominance of complex or radial tears involving the posterior horn (63.6%), whereas in those without dislocation, longitudinal tears predominated (85.7%), also affecting the posterior horn. The topographic distribution of medial and lateral meniscal tears according to dislocation status is illustrated in Fig. 3.

Regarding the lateral meniscus, complex or radial tears were more frequent in the dislocation group (73.3%), mainly located in the posterior horn (66.6%).

In the nondislocation group, longitudinal tears were again the most common pattern (80.0%), primarily affecting the posterior horn. The detailed topographic and morphological distribution of medial and lateral meniscal tears according to dislocation status is summarized in Table 3.

**Table 3:** Topographic and morphological distribution of medial and lateral meniscal tears

Meniscus/location	With dislocation (*n* = 35)	Without dislocation (*n* = 20)
Medial meniscus (*n* = 18)		
Anterior horn	Oblique (*n* = 1)	—
Body	Longitudinal (*n* = 3)	Bucket-handle (*n* = 1)
Posterior horn	Radial (*n* = 6); Complex (*n* = 1)	Longitudinal (*n* = 6)
Lateral meniscus (*n* = 20)		
Anterior horn	Radial (*n* = 2); Complex (*n* = 1)	—
Body	Radial (*n* = 1); Complex (*n* = 1); Horizontal (*n* = 1)	Oblique (*n* = 1)
Posterior horn	Radial (*n* = 4); Complex (*n* = 2); Longitudinal (*n* = 3)	Longitudinal (*n* = 4)

**Fig. 4:**
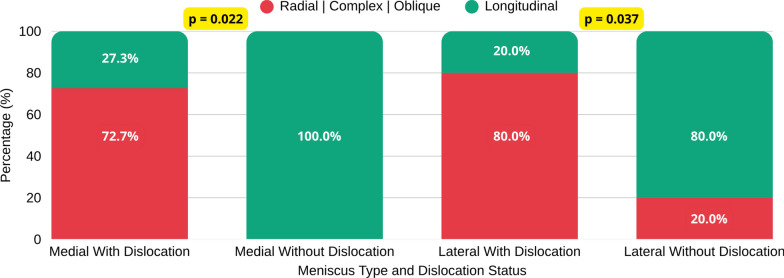
Morphological pattern of meniscal tears and knee dislocation

The association between the morphological pattern of meniscal tears and knee dislocation was statistically significant for both the medial (*p* = 0.022) and lateral meniscus (*p* = 0.037) as demonstrated in Fig. 4 and Table 4.

**Table 4:** Meniscal tear morphology according to dislocation status

*n* = 55 knees	With dislocation (*n* = 35)		Without dislocation (*n* = 20)		*p*-value
Lesion morphology	Radial, complex, or oblique	Longitudinal	Radial, complex, or oblique	Longitudinal	
Medial meniscus	8/35 (22,8%)	3/35 (8,5)	0/20 (0%)	7/20 (35%)	0.022*
Lateral meniscus	12/20 (60%)	3/20 (15%)	1/20 (5%)	4/20 (20%)	0.037*

^*^Significant at *p* < 0.05

These graphical trends are further detailed in Table 4, which summarizes the numerical distribution of meniscal tear morphologies according to dislocation status.

Univariate logistic regression analysis demonstrated a strong association between knee dislocation and meniscal tear morphology. Among patients without dislocation, all medial meniscal tears were longitudinal (7/7, 100%), whereas in those with dislocation, 72.7% (8/11) were radial, complex, or oblique (Fisher’s exact test, *p* = 0.004). This perfect separation indicates that dislocation is a strong predictor of nonlongitudinal tear morphology in the medial meniscus.

For the lateral meniscus, patients with dislocation had 16-fold higher odds of radial, complex, or oblique tears compared with those without dislocation (OR = 16.00, 95% CI 1.27–200.92; Fisher’s exact test, *p* = 0.031).

Post-hoc power analysis confirmed adequate statistical power (87.4%) to detect the observed effect for medial meniscus tears, with a large effect size (Cohen’s *h* = 1.502). For lateral meniscus tears, statistical power was 70.3%, also corresponding to a large effect size (Cohen’s *h* = 1.287).

These associations are summarized in Table 5, which demonstrates the strong relationship between knee dislocation and meniscal tear morphology. In the medial meniscus, perfect separation prevented odds ratio calculation due to all nondislocated cases presenting longitudinal tears, whereas patients with dislocation predominantly exhibited radial or complex patterns. For the lateral meniscus, the odds of nonlongitudinal tears were markedly higher in dislocated knees, even after adjustment for high-energy trauma and age.

**Table 5:** Association between knee dislocation and meniscal tear morphology

Meniscus	Comparison	Odds ratio (OR)	95% Confidence interval (CI)	*p*-value
Medial meniscus	Dislocation versus No Dislocation	Not calculable*	—	0.004**
Lateral meniscus	Dislocation versus no dislocation (Univariate)	16.00	1.27–200.92	0.031*
	Dislocation versus no dislocation (Adjusted) †	68.87 × 10^6^	0.00–∞	0.995

^*^Significant at *p* < 0.05

**Fisher’s exact test; perfect separation observed. In the nondislocation group, zero radial/complex/oblique tears were observed (all seven tears were longitudinal), precluding OR calculation via standard logistic regression

†Adjusted for high-energy trauma and age. Wide confidence intervals reflect small sample size and quasi-complete separation

## Discussion

In this study, medial and lateral meniscal tears were identified in 32.7% and 36.4% of knees, respectively. These rates are comparable to those reported in previous series of multiligament knee injuries. Kim et al. [11], in a systematic review and meta-analysis including 3391 patients, found pooled incidences of 30.4% for medial and 27.5% for lateral meniscal tears in MLKI. Krych et al. [12] observed meniscal tears in 55% of knees with dislocation, and Figueroa et al. [14] reported an incidence of 67.1% in MLKI. Thus, the overall frequency of meniscal injury in our cohort (32.7% medial, 36.4% lateral, 43.6% overall) falls within the previously described range of 29.4–67.1% [3–6, 11–14]. Beyond incidence, however, the main contribution of the present study is the demonstration of distinct morphological patterns of medial and lateral meniscal tears according to the presence or absence of knee dislocation.

For the medial meniscus, radial or complex tears were predominant in knees with dislocation, whereas longitudinal tears prevailed among patients without dislocation. Regarding the lateral meniscus, radial and complex tears—mainly involving the anterior and posterior horns—were more frequent in dislocated knees, while longitudinal patterns were the most common in nondislocated cases. These morphological differences, particularly in the medial meniscus, support the findings of Sim et al. [3], who also reported a higher prevalence of radial tears in dislocated MLKI and longitudinal tears in nondislocated knees. However, unlike Sim et al., who observed a predominance of anterior horn or completely detached lateral meniscus tears, our cohort showed a greater involvement of both posterior and anterior horns, with no higher incidence of complete detachment.

**Fig. 5:**
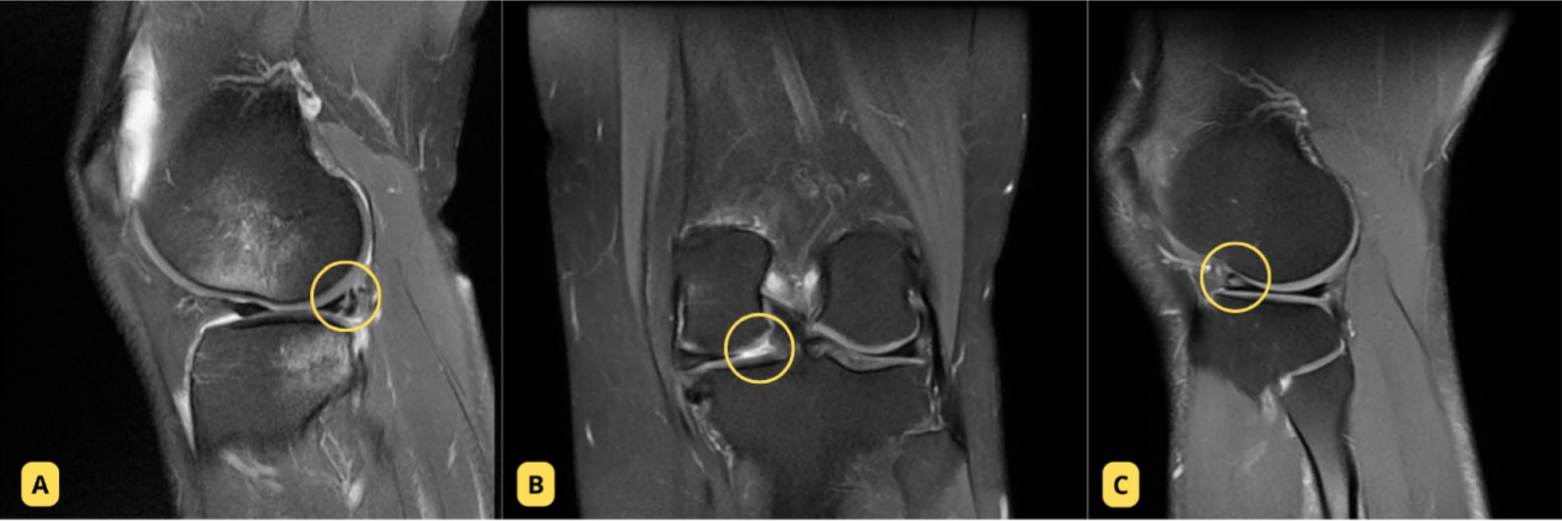
Representative magnetic resonance imaging (MRI) examples illustrating distinct meniscal tear morphologies. **A** Longitudinal tear of the posterior horn of the lateral meniscus in a nondislocated knee. **B** Radial tear of the posterior horn of the medial meniscus in a dislocated knee. **C** Complex tear of the anterior horn of the lateral meniscus in a dislocated knee

The biomechanical basis for these distinct patterns can be explained by the injury mechanisms and forces involved in knee dislocation. Anterior knee dislocations, which are the most common type, usually result from high-velocity hyperextension trauma [4, 19]. During hyperextension, anterior tibial translation combined with rotational stress leads to compressive and shear forces that concentrate at the posterior horn and root attachments of the menisci. These forces exceed the circumferential tensile strength of the collagen fibers, predisposing the tissue to radial or complex tears. The lateral meniscus, given its greater mobility and posterior excursion, is particularly susceptible to such high-energy shear forces. In contrast, lower-energy rotational injuries without frank dislocation typically generate traction along the circumferential fibers, resulting in longitudinal tear patterns. Representative MRI examples of these distinct morphological patterns are presented in Fig. 5.

The association between knee dislocation and radial or complex meniscal tears has important clinical implications. Recognizing these morphological patterns preoperatively can guide the arthroscopic approach and influence surgical sequencing. Complex or radial tears, particularly at the posterior horn or meniscal root, require specific repair techniques such as transtibial pull-out sutures or side-to-side repairs, which should be performed before cruciate ligament reconstruction to prevent graft impingement. Awareness of these tear configurations broadens the arthroscopic survey, reduces the likelihood of missed posterior horn lesions, and facilitates more accurate restoration of the meniscal function, potentially improving long-term outcomes after multiligament reconstruction. Furthermore, these findings can enhance the informed consent process, as patients with knee dislocations can be better advised about the higher likelihood of complex meniscal injuries requiring specialized repair techniques and potentially longer rehabilitation periods.

The analysis of meniscal tear distribution according to the Schenck classification demonstrated that more complex ligament injury patterns, such as KD IV and KD III-M, were associated with the highest absolute frequencies of meniscal tears, with 14 and 6 cases, respectively. These findings are consistent with previous reports suggesting that higher grades of ligamentous instability and multiplanar dislocation correlate with an increased risk of meniscal injury [3, 13, 14]. Interestingly, the KD I group—characterized by isolated cruciate ligament injury combined with collateral involvement—also showed a considerable incidence of meniscal tears (*n* = 10), indicating that even less complex ligament injury patterns can produce significant overload on the meniscal structures. While some studies have suggested that meniscal tears are more common in isolated ACL injuries than in MLKI [14], others have shown the opposite, particularly when ACL and MCL injuries occur together, which may represent a more aggressive pattern for the medial meniscus [13].

The KD III-L group, which involves posterolateral corner disruption, accounted for only three documented meniscal tears. However, this number is likely underestimated, as MRI has limited sensitivity for detecting posterolateral corner injuries in MLKI, with diagnostic failure rates reported in up to 80% of cases [20, 21]. In the KD V group, which includes periarticular fractures, the incidence of meniscal tears was 42.8% (3 of 7 cases), all associated with tibial plateau fractures. When only this fracture type was considered, the concomitant meniscal injury rate increased to 75%, aligning with previously published data that report a wide range between 21 and 90% [19, 20].

In addition, all neurovascular and soft-tissue injuries in this cohort occurred in association with knee dislocation and high-energy trauma. Two cases (3.6%) of popliteal artery injury were identified, consistent with the 3.3–64% incidence reported in the literature [6, 7]. Five cases (9.1%) of common peroneal nerve injury were also observed, exclusively in dislocated knees. This pattern has been widely described, particularly in dislocations involving the posterolateral corner (KD III-L and KD IV), with reported incidences ranging from 25 to 40% [21, 22]. These findings reinforce that knee dislocation not only involves extensive ligamentous disruption but also carries a substantial risk of neurovascular and soft-tissue injury, further highlighting the severity of high-energy trauma mechanisms in MLKI.

This study has several limitations that should be considered when interpreting its findings. First, its retrospective design relies on medical records and MRI assessments, which introduces potential information bias and dependence on imaging accuracy for ligamentous and meniscal evaluation. Second, all patients were treated at a single tertiary trauma center, which may limit the generalizability of these results to other populations or less severe MLKI presentations. Third, MRI examinations were performed using 1.5 T scanners with 4.5-mm slice thickness, which may have limited the detection of subtle root or radial tears. Although all images were independently reviewed by a musculoskeletal radiologist and an orthopedic knee specialist, inter-reader reliability was not quantitatively assessed. While the post-hoc power analysis suggested adequate statistical power (87.4% for medial meniscus and 70.3% for lateral meniscus), we acknowledge that such analyses are inherently circular when calculated from observed effects and should be interpreted with caution. Ideally, sample size should be determined a priori on the basis of clinically meaningful effect sizes. Finally, the absence of arthroscopic correlation or postoperative outcomes precludes validation of MRI findings against intraoperative observations and functional recovery.

## Conclusions

This study demonstrates that knee dislocation is strongly associated with a distinct and more destructive pattern of meniscal tears compared with nondislocated multiligament knee injuries. This difference is characterized by a higher prevalence of radial, complex, and oblique tears in the dislocated group. Notably, a perfect separation was observed in the medial meniscus, with all nondislocated cases presenting longitudinal tears (*p* = 0.004), and patients with dislocation showing 16-fold higher odds of complex lateral meniscal tears (OR = 16.00, 95% CI 1.27–200.92). Recognition of these morphological patterns is essential for accurate diagnosis, comprehensive arthroscopic assessment, and surgical planning aimed at optimizing outcomes in multiligament knee reconstruction.

## Data Availability

The datasets generated and/or analyzed during the current study are not publicly available due to patient confidentiality but are available from the corresponding author on reasonable request.
